# An Innovative Cascade System for Simultaneous Separation of Multiple Cell Types

**DOI:** 10.1371/journal.pone.0074745

**Published:** 2013-09-06

**Authors:** Arkadiusz Pierzchalski, Anja Mittag, Jozsef Bocsi, Attila Tarnok

**Affiliations:** 1 Translational Centre for Regenerative Medicine (TRM), University of Leipzig, Leipzig, Germany; 2 Department of Pediatric Cardiology, Heart Center GmbH, Faculty of Medicine, University of Leipzig, Leipzig, Germany; 3 LIFE - Leipzig Research Center for Civilization Diseases, Universität Leipzig, Leipzig, Germany; University of São Paulo, Brazil

## Abstract

Isolation of different cell types from one sample by fluorescence activated cell sorting is standard but expensive and time consuming. Magnetic separation is more cost effective and faster by but requires substantial effort. An innovative pluriBead-cascade cell isolation system (pluriSelect GmbH, Leipzig, Germany) simultaneously separates two or more different cell types. It is based on antibody-mediated binding of cells to beads of different size and their isolation with sieves of different mesh-size. For the first time, we validated the pluriSelect system for simultaneous separation of CD4+- and CD8+-cells from human EDTA-blood samples. Results were compared with those obtained by magnetic activated cell sorting (MACS; two steps -first isolation of CD4+, then restaining of the residual cell suspension with anti-human CD8+ MACS antibody followed by the second isolation). pluriSelect separation was done in whole blood, MACS separation on density gradient isolated mononuclear cells. Isolated and residual cells were immunophenotyped by 7-color 9-marker panel (CD3; CD16/56; CD4; CD8; CD14; CD19; CD45; HLA-DR) using flow cytometry. Cell count, purity, yield and viability (7-AAD exclusion) were determined. There were no significant differences between both systems regarding purity (MACS (median[range]: 92.4% [91.5-94.9] vs. pluriSelect 95% [94.9-96.8])) of CD4+ cells, however CD8+ isolation showed lower purity by MACS (74.8% [67.6-77.9], pluriSelect 89.9% [89.0-95.7]). Yield was not significantly different for CD4 (MACS 58.5% [54.1-67.5], pluriSelect 67.9% [56.8-69.8]) and for CD8 (MACS 57.2% [41.3-72.0], pluriSelect 67.2% [60.0-78.5]). Viability was slightly higher with MACS for CD4+ (98.4% [97.8-99.0], pluriSelect 94.1% [92.1-95.2]) and for CD8+-cells (98.8% [98.3-99.1], pluriSelect 86.7% [84.2-89.9]). pluriSelect separation was substantially faster than MACS (1h vs. 2.5h) and no pre-enrichment steps were necessary. In conclusion, pluriSelect is a fast, simple and gentle system for efficient simultaneous separation of two and more cell subpopulation directly from whole blood and provides a simple alternative to magnetic separation.

## Introduction

Cell separation methods are widely used in cell biology, immunology and oncology. They enrich or isolate cells based on the phenotypic or functional features of different cell types such as differences in size, shape (morphology), cell membrane, cytoplasmic or cell nucleus composition or other characteristics. In general, cell separation methods can be grouped into the following categories.

1. Physical separation techniques – density gradient centrifugation, counterflow elutriation or filtration separate cells due to their density and size differences. By setting the centrifuge to spin at various speeds or by establishing different density gradients, cells of different masses and densities can be isolated. Physical separation methods are valuable first stage methods for separation of different cell types [1–3] or removing large amount of cells from the sample but not affecting the target cells [4]. Advantages are that these methods are label free, and relatively fast, and that they can be used for large numbers of cells. However, they have limited specificity, thus specific cell types cannot be isolated. High cell specificity can be obtained by erythrocyte rosetting [5,6] in combination with density gradient centrifugation.
2. Fluorescent antibody-based cell sorting – is the method of choice to isolate cells based on multiple cell characteristics and is performed on a Fluorescence-Activated Cell Sorter (FACS), a specialized type of flow cytometry, by droplet sorting. The cell sorter was invented by Mack Fulwyler in 1965 [7] and further improved for fluorescence applications [8,9]. It provides fast, objective and quantitative recording of fluorescent signals from individual cells as well as physical separation of cells of particular interest [10]. FACS can simultaneously sort different cell types into two or more containers, one cell at a time, based upon their light scattering and fluorescence pattern. However, it needs large investment, is relatively slow when high numbers of cells with a high purity are needed and aerosol formation by the droplet sorting may render a risk [11]. Microfluidic cell sorters avoid aerosol borne risk but are mostly slower than FACS and allow sorting of one cell population only [12].
3. Magnetic antibody-based cell-isolation - this method is based on antibody tagging of cells with a tiny iron bead. The cells are then separated in a magnetic column retaining the bead bearing cells in the magnetic field [13,14]. High cell numbers can be isolated rapidly. Positive selection, by labeling the target cells, is the fastest and the most efficient way to isolate a cell subset with high purity and yield. A negative selection is needed when the cells of interest have to be “untouched” for subsequent analyses or the specific antibody is non-available for the cell-subtype (15). Hence, all the cells which need to be removed from the sample have to be tagged with a magnetic bead. Because separation is based on a single parameter (i.e., magnetization), this method is generally effective only for the isolation of a single cell population. Different cell populations can be isolated from a single sample by sequential magnetic sorting. This approach is however time consuming and laborious and requires in the case of higher yield isolation from whole blood density gradient isolated leukocytes. Recently Miltenyi Biotec GmbH (Bergisch Gladbach, Germany) has introduced a whole blood magnetic beads separation which is however limited by column capacity up to 15 ml blood volume [16].

Most widely used for the isolation of specific cell types are FACS and MACS and selection of the right separation techniques depends on the question raised (17). However, a number of investigations have pursued multiparameter, multitarget magnetic separation methods to combine the advantages of screening and selection techniques. For example, Chalmers et al. [18] achieved separation of cells based on their degree of magnetic labeling by using conventional macro scale magnetic dipoles to generate high magnetic field gradients. This approach achieved a high level of purity but suffered from relatively low throughput. Adams et al. [19] introduced microfluidics technology called MT-MACS to achieve simultaneous spatially-addressable sorting of multiple target cell types in continuous-flow. By combination of two different magnetic tags with distinct magnetization and size two cell types can be sequentially isolated. This device is well suited for sorting targets that are smaller or comparable in size to bacteria limiting its application for mammalian cells. Both above mentioned approaches are still in an early phase of development and require additional sophisticated equipment for cell separation.

Recently, pluriSelect GmbH (Leipzig, Germany) introduced an innovative, pluriBead - Cascade system which relies on simultaneous physical separation of two or more different cell types. It is based on antibody-mediated binding of cells to beads of two or more different diameters (much bigger than cell size) each type labeled with a different antibody. These beads and the attached cells are then isolated with sieve cascade of different mesh-size. Non bound cells go through the mesh because the mesh diameter is much bigger than the cell size. By this easy to handle system a high number of different cell types can be separated in a single step from whole unseparated blood from practically infinite blood volume. In this paper we present for the first time a validation of the pluriSelect system for the simultaneous separation of CD4+ and CD8+ cells in comparison to MACS magnetic isolation.

## Material and Methods

### Sample collection

The use of blood samples from healthy human volunteers for developing and establishing blood based assays was approved by the Ethical Committee of the Faculty of Medicine at the University of Leipzig, Germany. Whole venous blood samples were drawn from 11 healthy adult volunteers (age: 30-48) from Leipzig who have signed an informed written consent.

### pluriSelect cascade isolation

**Figure 1:**
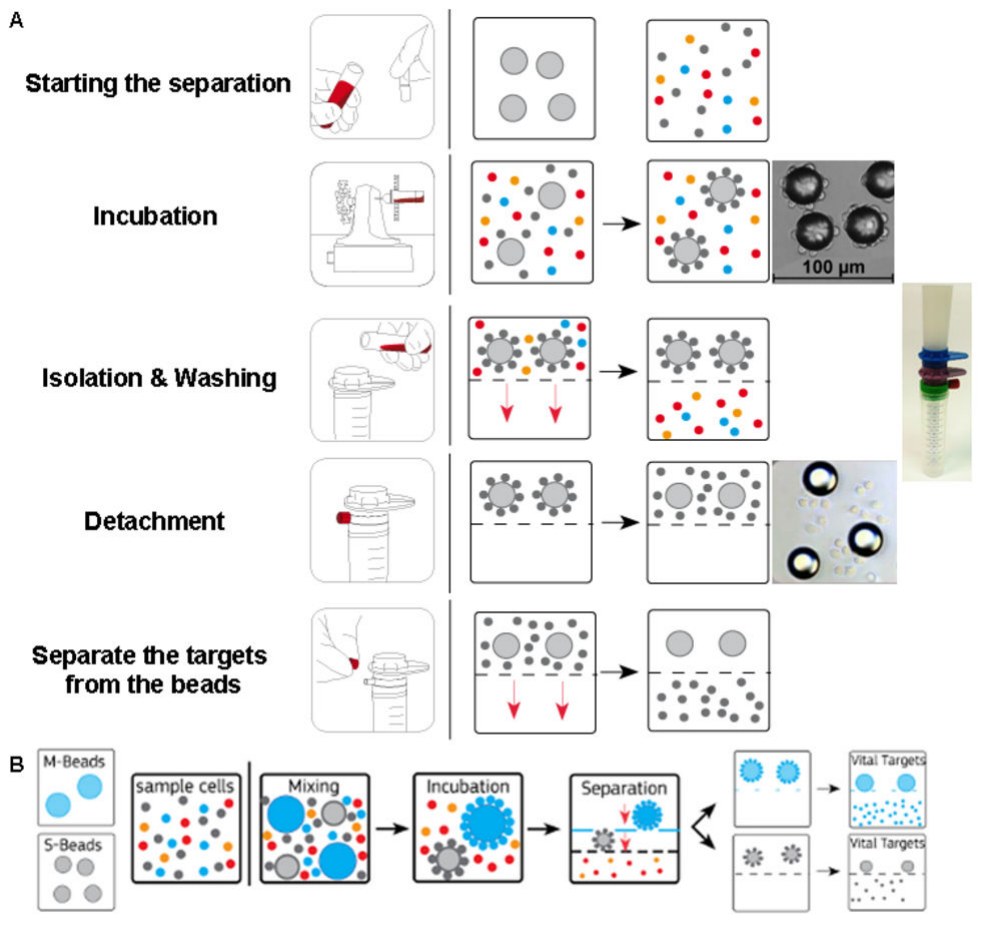
Overall overview of pluriSelect cascade cell separation. A. Scheme showing the principle of pluriSelect cell separation. B. Scheme showing overview of cascade cell separation. For the description see the text.

**Figure 2:**
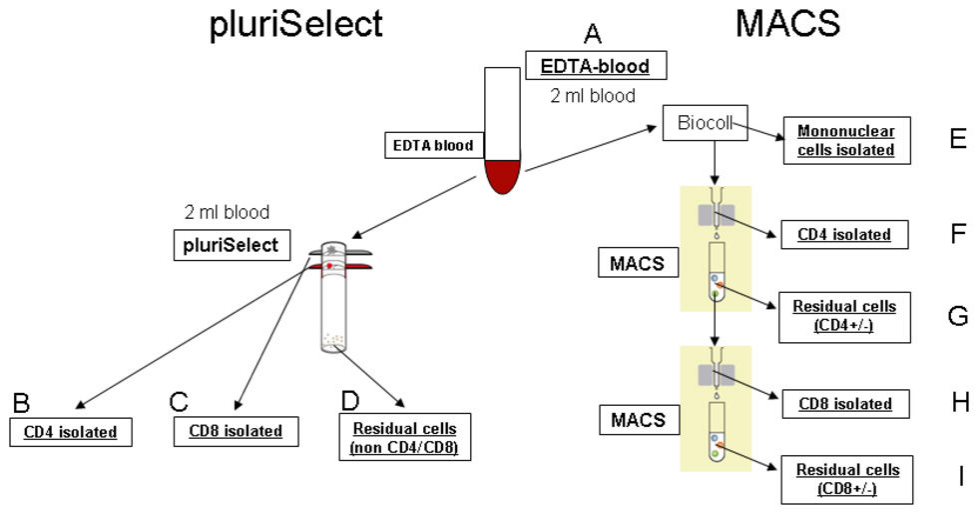
Workplan summary. The original blood sample (A) was aliquoted for the pluriSelect and the MACS isolation procedure. Without further preparations the EDTA blood was subjected to the pluriSelect cascade system (left column, samples B–D). For the MACS system PBMC were isolated prior to the separation steps (right column, samples E-I). From each preparations (A–I) aliquots were taken, analyzed, and compared between both systems. The analysis included cell counting, viability assessment and immunophenotyping.

Figure 1 shows a schematic step-by-step overview of the isolation process for one bead type (Figure 1 part A) and mixture of two different bead types (Figure 1.B). Figure 2 shows schematically the nine different samples (A-I) collected and further analyzed for pluriSelect and MACS. The pluriSelect cascade isolation of CD4+ and CD8+ cells was done directly from whole blood. For this purpose, 2 ml EDTA blood (sample A) was incubated with antibody-coated beads (pluriBead^R^) with two different bead sizes (S-size, 30µm diameter, specific for anti-human CD4, antibody clone: MEM-241, catalog No. 10-00400-11; M-size, 60µm diameter, specific for anti-human CD8, antibody clone: MEM-31, catalog No. 10-00800-21) in a mixing container at room temperature for 30 min on a horizontal pluriPlix^R^ roller (8 rpm). In initial experiments we tested different incubation times (20-60 min) and found 30 min incubation optimal with regard to yield and viability. At shorter incubation yield was lower at longer incubation yield slightly increased but cell viability was compromised. Next, the cell–bead mixture was pipetted on a sieve cascade provided with the sieve with the larger mesh holes on top of the sieve with the smaller ones. Thereby, the larger beads are sieved out by upper sieve while smaller beads are passing through and are sieved out by the second sieve. For increasing the purity the cells (attached to the beads) were washed on the sieve thoroughly with ~45 ml wash buffer (PBS w/o Ca^2+^ and Mg^2+^ pH 7.4 with 2mM EDTA and 0.5% BSA) (Sigma-Aldrich, St. Louis, MO, USA) to remove erythrocytes, unbound cells, and the small beads onto the second sieve. Only the cells attached to the small beads were retained by the second sieve. Non-target cells were washed through the sieve and hence removed from the system. After washing, each sieve was put onto a separate sterile 50 ml centrifuge tube (catalog No. 91050; TPP, Trasadingen, Switzerland) using a connector ring. The Luer-Lock of the adapter was closed to stop the flow though the mesh and 2 ml detachment buffer was put on top of each sieve in order to achieve a release of the cells from the beads. The sample was incubated for 10 min at room temperature. Cells were separated from the beads by careful aspiration by pipette. The Luer-Lock was opened and the detachment buffer with the released cells ran into the centrifuge tube through the sieve. For increasing the yield beads were washed on the sieves with additional 8 ml wash buffer. Separated CD4+ (Sample B) and CD8+ (sample C) cells as well as the residual wash-through (sample D) were centrifuged for 10 min at 250g and resuspended in 1 ml wash buffer for further analysis.

### Magnetic step by step isolation by MACS method

CD4+ and CD8+ cells were isolated via MACS microbead system using miniMACS columns (Miltenyi Biotec GmbH). The system does not allow for the simultaneous separation of different cell types so that the isolation was done in two consecutive steps. Mononuclear cells (PBMC) from 2 ml EDTA- anticoagulated whole blood were isolated by density gradient centrifugation using Biocoll (AG Biochrom, Berlin, Germany). PBMC were washed twice in wash buffer (PBS w/o Ca^2+^ and Mg^2+^ pH 7.4 with 2mM EDTA and 0.5% BSA) (sample E).

Step 1. CD4+ cell isolation. Cells were resuspended in 80 µl wash buffer and 20 µl of anti-human CD4 specific MicroBeads were added. After 15 min incubation at 4°C cells were washed in 1.5 ml wash buffer and centrifuged at 300*g* for 10 min. Cells were resuspended in 500 µl wash buffer. Magnetic separation: Cell suspension was filled into the prerinsed MS column in the magnetic field of the MACS magnet, washed 3 times with wash buffer. The magnetic labeled CD4+ cells were bound to the column. The non-labeled and CD4+ depleted wash-through cells were collected for the second separation step (sample G CD8+ cells labeling and separation). The column was removed and the magnetic labeled CD4+ cells were released from magnetic field. The column was washed out through with 1 ml wash buffer and collected for further analysis (sample F).

Step 2. CD8+ cell isolation the wash-through cells from step 1 (Figure 2 G) were centrifuged at 300g for 10 min. The supernatant was discarded to remove the anti-human CD4 specific microbeads, cells were resuspended in 80 µl wash buffer, and 20 µl of anti-human CD8 specific MicroBeads were added. After 15 min incubation at 4°C cells were washed with 1.5 ml wash buffer and centrifuged at 300g for 10 min. Cells were resuspended in 500 µl wash buffer and the magnetic separation was done as described in the Step 1. The positive selected CD8 cells (sample H) as well as CD8 depleted wash through fraction (sample I) were subjected to further analysis.

### Immunophenotyping

All of above collected nine aliquots from the preparations (A-I) were processed for cell analysis (detailed in [20]). Immunophenotyping was done in a one-tube assay with the following anti-human antibodies: CD3-FITC (BD Biosciences, clone: SK7); CD16/56-PE (BD Biosciences, clone: B73.1/MY31); CD4-PE-Cy7 (eBiosciences, clone: RPA-T4); CD8-APC (Beckman Coulter, clone: B9.11); CD14-APC-H7 (BD Biosciences, clone: MϕP9); CD19-APC (Immunotools, clone: LT19); CD45-PacBlue (BD Biosciences, clone: Hl30); HLADR-PerCP (BD Biosciences, clone: L243). 50 µl of each preparation (A-I) was stained for 1h at room temperature in the dark. Subsequently, erythrocytes were lysed and the samples were fixed in Versalyse lysing solution (Beckman Coulter, USA) for 8 min. The samples were analyzed on CyFlow ML Partec flow cytometer (Partec GmbH, Münster, Germany) equipped with 3 lasers – blue 488 nm; red 645 nm; violet 405 nm; filter settings: FL1 (FITC) -527/30nm BP (Bandpass); FL2 (PE) -590/30nm BP; FL3 (PerCP) -682/30nm BP or FL3 (PE-Cy7) -680nm LP (Longpass); FL4 (APC) -675/20nm BP; FL5 (APC-H7) -748nm LP; FL6 (PacificBlue) -455/30nm BP.

**Figure 3:**
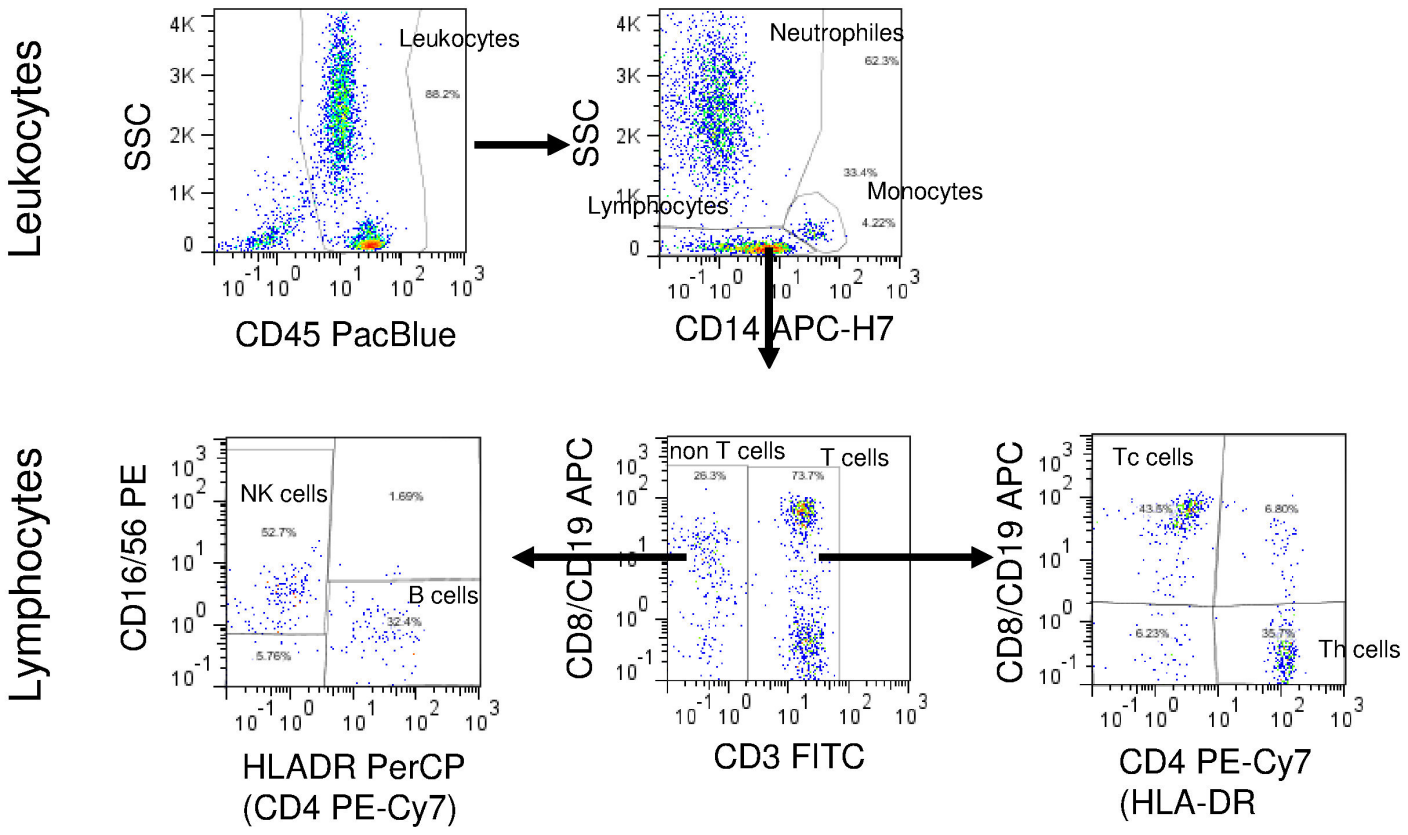
7-color, 9-markers immunophenotyping analysis scheme by flow cytometry. The different leukocyte subtypes were analyzed by the shown gating strategy. In all CD45 positive leukocytes the following cells were determined: CD3 (lymphocytes); CD4 (Th lymphocytes); CD8 (Tc lymphocytes); CD19 (B lymphocytes). Leukocytes small differential picture was done using CD14 and SSC display. In lymphocytes CD3 positive and negative (non-T cells) have been identified. In T cells CD4 and CD8 positive cells were estimated. In non-T cells NK or B cells (based on CD16/56 or HLADR expression, respectively) were identified. Proportions of the analyzed cell types were determined and the respective absolute cell numbers of these cell types were calculated by the absolute cell counts.

Flow cytometric analysis was done as shown in Figure 3. All cells with CD45 expression and appropriate side scatter (SSC) were identified as leukocytes. Using CD14 marker and SSC lymphocytes, monocytes and neutrophils were identified. Cell numbers of T lymphocytes (CD3 positive), T helper cells (Th, CD4 positive), T cytotoxic cells (Tc, CD8 positive), non-T cells (CD3 negative), B lymphocytes (CD19 positive), and NK cells (CD16/56 and CD16/56/8) in leukocytes and in lymphocytes were determined. NK cells were discriminated based on their CD16/56 and lack of HLA-DR expression. Original list-mode data files and FlowJo analysis files are available through the public FlowData repository (http://flowrepository.org/) Data set name: pluriSelect under the following link: http://flowrepository.org/id/RvFrmVgMAmzXCUzeMqfssRsuO5wCWqQWJi7E8MaIyk3iU2Wlyv2Hgwg3aazEoqzy.

### Cell count

Cell counts from whole blood and the residual pluriSelect blood samples were measured on Sysmex KX-21 (Kobe, Japan) hematology analyzer. The count for all positively isolated cells, Biocoll gradient isolated samples, and MACS residual samples was determined on Z2-Coulter Counter (Beckman Coulter, USA). The cell counts of leukocytes served as reference for the calculation of the yield and cell loss as well as cellular contamination.

### Cell viability

CD4 and CD8 positively selected cells were stained with CD3 FITC for 30 min, afterwards counterstained with 7-AAD (5 µg/ml) for 5 min and analyzed by CyFlow ML. Samples other than positively isolated cells were stained as described in immunophenotyping, followed by 7-AAD staining (5 µg/ml). Hence, the viability of monocytes and neutrophils was estimated and the initial CD4+ and CD8+ viability too.

### Analysis software and statistical analysis

For each analyzed cell population yield (Y) and purity (P) was calculated as follows:

Y[%]=100 * (cell number (isolated)) / (cell number (whole blood))

P[%]=100 *(cell number (target cells isolated)) / (cell number (all cells isolated))

All data have normal distribution as determined by Kolmogorov-Smirnov test. Statistical significance for the differences between both separation systems was estimated using two-sided paired student’s t-test. Data were regarded statistically significant if p<0.01. P value threshold was corrected by Bonferroni in multiple comparisons (SPSS Statistics ver. 19.0.1, IBM Corporation, New York, USA).

## Results

### 1: Yield and purity

**Figure 4:**
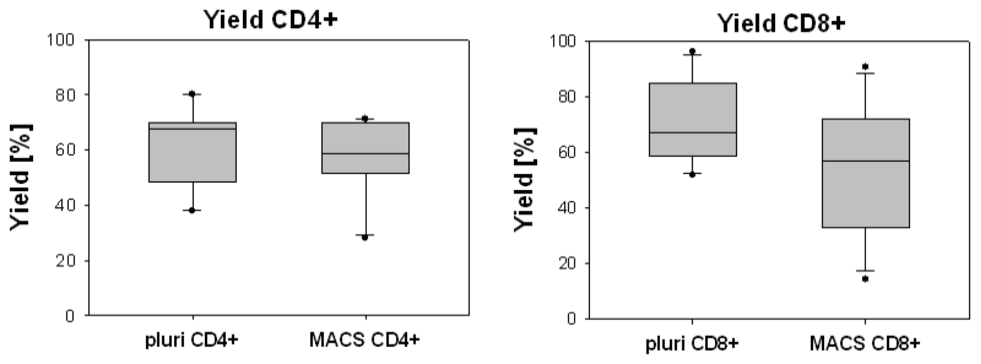
Yield obtained by pluriSelect and MACS isolation. The values are given as median 25-75% range IQR (box), Whisker (5-95%) and dots (outliers). Statistical significance by student’s T-test for p<0.01 (Bonferroni corrected significance) (n=11).

We obtained a slightly higher yield for CD4+ and CD8+ cells isolated by pluriSelect (Figure 4). These differences were however not significant (CD4+ cells (median [range]): 67.9% [56.8-69.8] vs. 58.6% [54.1-67.5], CD8+ cells: 67.2% [60-78.6] vs. 57.2% [41.3-72]).

**Figure 5:**
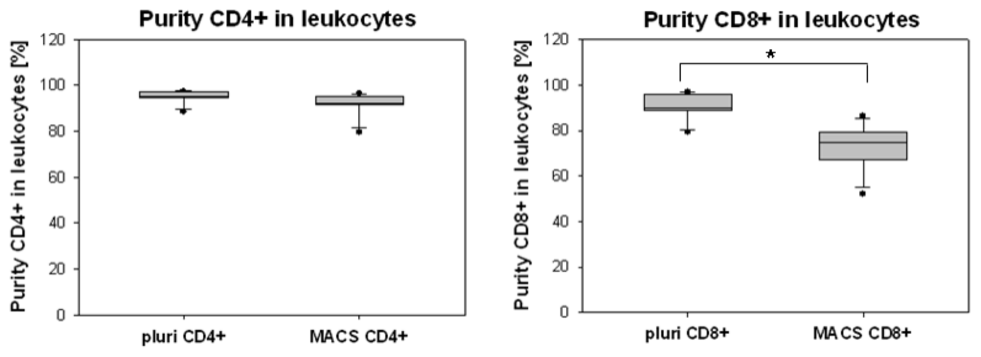
Purity of CD4+ and CD8+ samples after pluriSelect and MACS isolation. The values are given as median 25-75% range IQR (box), Whisker (5-95%) and dots (outliers). Statistical significance by student’s T-test for p<0.01(Bonferroni corrected significance).

As shown in Figure 5 the purity of CD4+ isolated cells was comparable for both systems. However, for the CD4+ cells there was significantly lower content of contaminating cells after pluriSelect isolation compared to MACS (95.0% [94.9-96.1] vs. 92.4% [91.5-94.9]), respectively. The purity for CD8+ isolated cells by pluriSelect was 15% higher than by MACS isolation (89.9% [89.0-95.7] vs. 74.8% [67.6-78.0]).

### 2: Determination of cell type specific contamination

**Figure 6:**
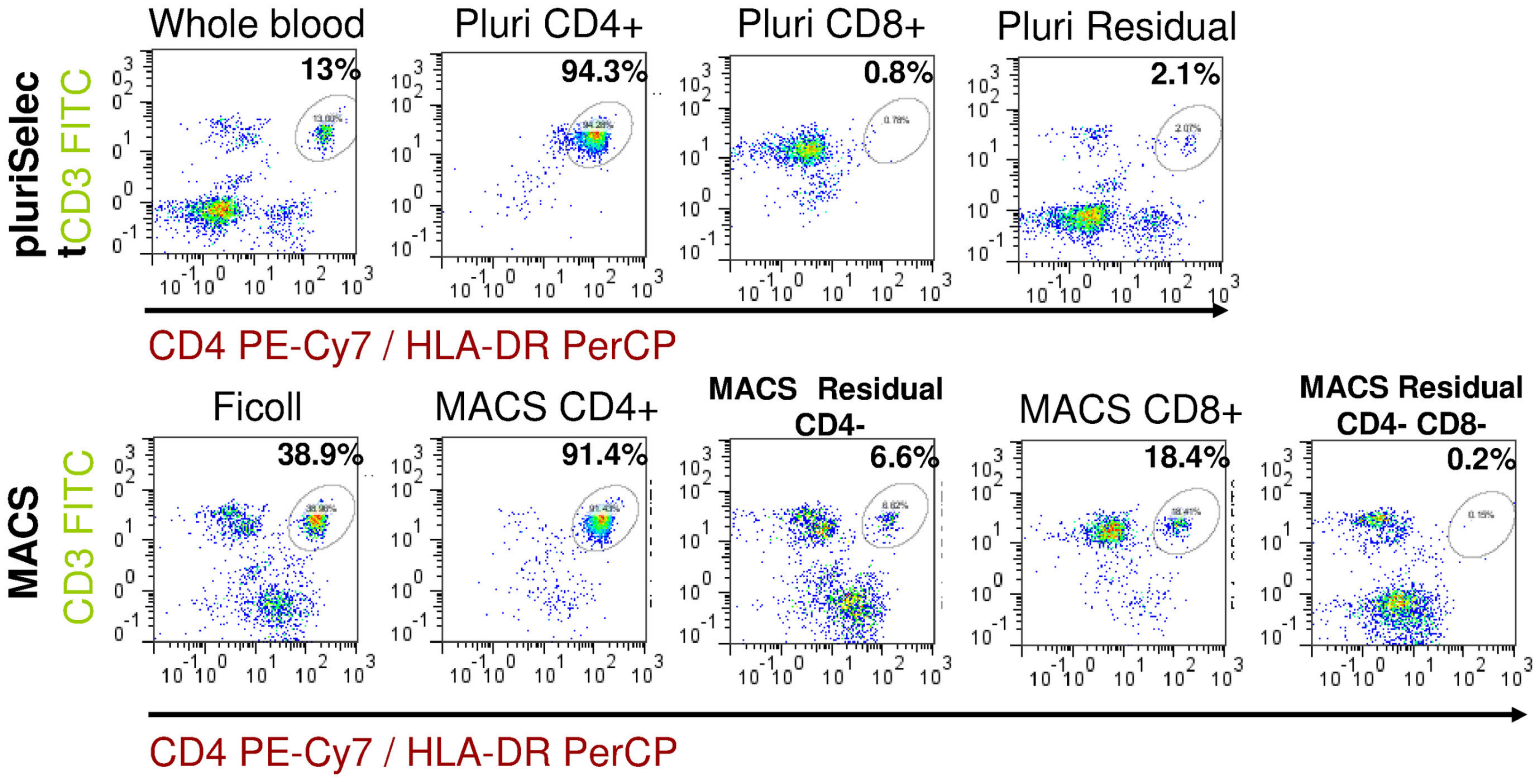
Representative CD3 vs. CD4 plots. The plots show the efficiency of CD4+ isolation by pluriSelect A-D and MACS E-I. % - percent of CD4+ in different samples for comparison of gain and the loss of CD4+ during cell separation. The circle shows the presence of CD4+ cells in different samples. CD4+ represents the main impurity of CD8+ sample (H).

The clearly lower purity of CD8+ cells in the MACS isolated samples (Figure 2. sample H) suggested a contamination of the CD8+ cells with other cells. Figure 6 shows CD3 vs. CD4 dotplots for all isolated samples (Samples A-I). In pluriSelect CD8+ isolation samples (sample C) there was only a minor contamination with CD4+ cells. In MACS isolation, however, there was a substantial contamination of CD4+ cells within the CD8+ isolated samples (sample H) but almost no other cell type.

We also evaluated other contaminating cells. The focus was on monocytes bearing also CD4 on their cell surface. As shown in Table 1 there was a higher proportion of monocytes in the CD4+ MACS isolated samples (sample F) compared to the pluriSelect isolated CD4+ samples (sample B; 3.5% vs. 0.8%). Moreover, there was a higher number of monocytes in the MACS CD8+ isolated samples (sample H) than in the CD8+ pluriSelect isolated samples (sample C; 3.45% vs. 0.2%).

**Table 1:** Characterization of contaminating leukocytes.

**Samples, **see Figure **2**	**A. Initial whole Blood Sample**	**B. CD4+ pluriSelect**	**C. CD8+ pluriSelect**	**F. CD4+ MACS**	**H. CD8+ MACS**
**Monocytes (CD14^+^ SSC^mid^) % of leukocytes**	6.86 (5.93-8.22)	0.80 (0.50-2.00)	0.20 (0.16-0.29)	3.50* (2.30-4.10)	3.45* (2.45-4.90)
**NK cells (CD16/56+ and CD16/56/8) % of lymphocytes**	13.72 (10.26-17.16)	0.11 (0.05-0.19)	2.29 (1.01-5.73)	0.72* (0.59-1.35)	3.02 (1.45-3.98)
**B lymphocytes (CD19+) % of lymphocytes**	14.12 (9.73-16.22)	0.33 (0.14-0.52)	0.12 (0.08-0.18)	0.85* (0.55-1.13)	1.20* (1.02-1.90)

Table shows the summary for determination of contaminating leukocytes (n=11). Data show median and range in parenthesis. * shows significant differences between values obtained by MACS and pluriSelect (Sample F vs Sample B or Sample H vs. Sample C from Figure 2). It was regarded statistically significant if p<0.01 (Bonferroni corrected significance).

Next, we estimated the number of NK contaminating cells (CD16+/56+), of which some are CD8+ or CD4+ but CD3-. In pluriSelect and MACS isolated CD8+ cell samples (samples C and H) we found similar numbers of NK cells, 2.3% vs. 3.0%, respectively. The difference was not statistically significant. However, there were statistically significantly higher proportions of NK cells (0.7%) in MACS CD4+ isolated samples (sample F) than in the pluriSelect CD4+ samples (sample B) 0.1%.

B lymphocytes (CD19+, HLA-DR+) were also present as contaminating cells in both the CD4+ and the CD8+ samples. There was a significantly higher number of B lymphocytes in both samples isolated by MACS as compared to pluriSelect (CD4+ samples F vs. B: 0.9% vs. 0.3%; CD8+ samples H vs. C: 1.2% vs. 0.1%)

### 3: Viability and time validation

**Figure 7:**
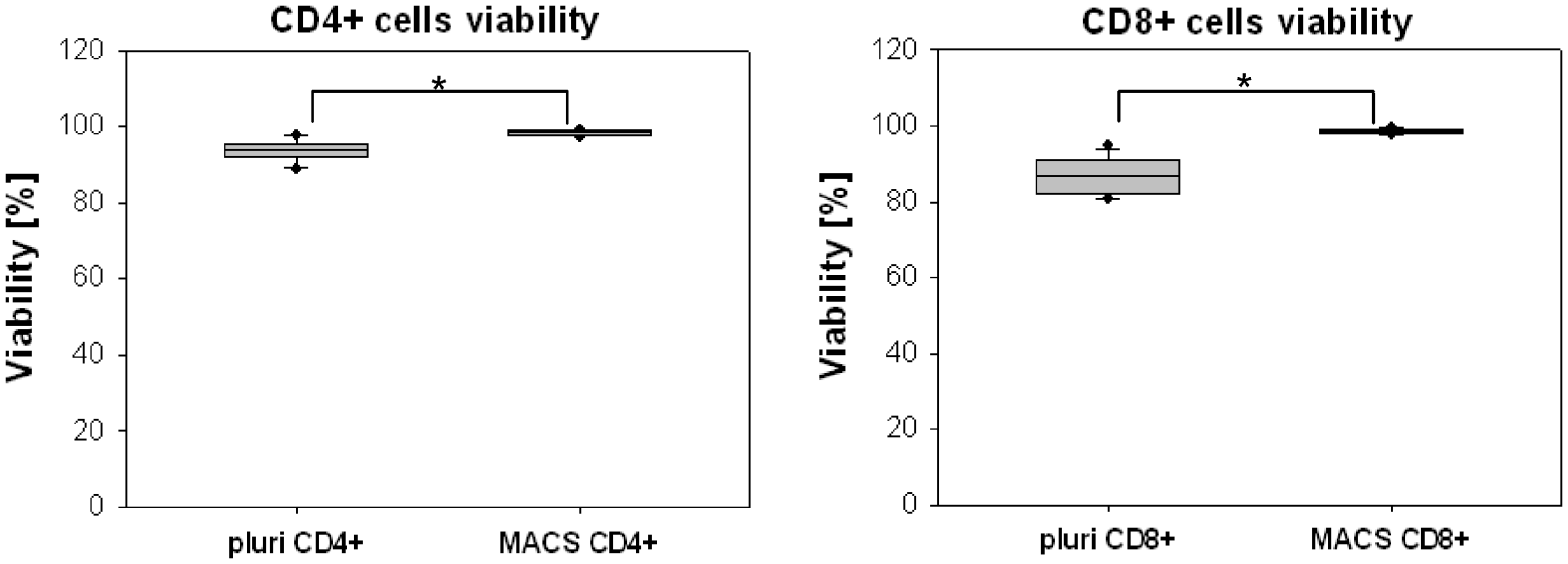
Cell viability of CD4+ and CD8+ samples after pluriSelect and MACS isolation. For both cell types the viability was very high. Isolation by MACS resulted in slightly higher cell viability than pluriSelect. In both cases this difference was statistically significant by Student’s t-test for p<0.01 (Bonferroni corrected significance) (n=11).

As shown in Figure 7 the viability of CD4+ cells was statistically significant lower for pluriSelect isolated cells than for MACS isolated cells (94.1% [92.1-95.2] vs. 98.4% [97.8-99.0]). In case of CD8+ isolated cells there was lower cell viability for pluriSelect compared to MACS (86.7% [84.2-89.9] vs. 98.8% [98.3-99.1]). Moreover, the cell viability of the two main residual cell populations (monocytes and neutrophils) was not affected by pluriSelect cell isolation (97.1% [95.6-98.7] vs. 94.1% [87.6-96.6]) compared to the cell viability of these cells in whole blood (98.7% [97.9-99.3] vs. 97.4% [91.7-98.8]). In case of MACS isolation there was statistically significant lower cell viability for monocytes after Biocoll density gradient isolation (93.3% [86.6-94.5]). Monocytes viability was yet lower after CD4+ MACS isolation (86.5% [82.6-92.3]) and decreased more after subsequent CD8+ MACS isolation (83.5% [69.6-92.3]). We also observed a lower cell viability of neutrophils upon Biocoll density gradient isolation (88.2% [82.4-89.1]). The viability of neutrophils decreased after CD4+ MACS isolation (80.3% [74.0-87.5]) and was higher after CD8+ MACS isolation (88.7% [84.6-95.3]). Isolation times using pluriSelect were with ~60 minutes much shorter than the ~145 minutes needed for the MACS step-by-step approach.

## Discussion

Among many cell isolation methods the bead-based methods belong to those that have revolutionized the cell separation world and ease the life of cell biologists [14] and cell therapists. Here we present a new separation principle which is also bead-based but in contrast to other methodologies for example the widely used magnetic labeling, it employs sieves for separation. The main advantages of this new method are (1) that it is designed to isolate the cells directly from the whole blood, i.e. no pre-enrichment or cell elimination steps are required prior to cell isolation and (2) it allows separation of not only one but two cell types in one cycle. No matter how many cell types are isolated simultaneously the separation cycle lasts only 1 hour. Here we tested the pluriSelect system in comparison to the well-established MACS separation for the isolation of CD4+ and CD8+ cells.

In our hands the pluriSelect system worked well, giving good cell yields, high purity and high viability comparable to the ones obtained by MACS. The yield of about 70% of the initial number of CD4+ or CD8+ cells in the whole blood can be expected by bead-based isolation according to the literature [21]. As there is almost 100% bead recovery in the isolation procedure, the yield may still be increased by applying more beads per sample. We started with about 300,000 CD4 specific S-size beads and 75,000 CD8 specific M-size beads for two ml of blood and recovered 280,000 and 63,000, respectively. In theory an S-size bead can bind up to 28 lymphocytes and the M-size bead up to 100 lymphocytes when they are completely covered by captured cell. In our experiments we found that an S-size bead bound 3-6 CD4+ cells and the M-size bead 3-16 CD8+ cells. Furthermore, the number of cells per bead increased with cell density of the target cells in the blood sample (not shown). The number of cells bound per bead depends on stochastic processes including probability of physical contact of cells with beads (cell/bead ratio in the suspension), available free space on the bead and on and off rate of bound cells. By increasing bead numbers per sample the yield can only be slightly increased as the number of cells bound per bead is in turn reduced as long as the overall binding capacity of the beads is higher than the target cell number. This, it seems that the bead numbers we used in our experiments is near to optimal conditions also in relation to cost effectiveness. It is also noteworthy, that in contrast to using soluble antibodies there is no dilution as the catching antibodies are immobilized on the beads. Thereby, the method allows designing your experimental setup in order to yield a defined number of target cells and it is hardly possible that the sieves are overloaded by a very high number of cells in the starting sample.

We have shown that the pluriSelect system is clearly more convenient than MACS if two or more cell types are to be isolated. This makes pluriSelect system superior compared to magnetic beads-based separation systems. Although more than one cell type can be labeled simultaneously by different antibodies coupled to ferromagnetic particles, it is not possible to differentiate and separate them magnetically [14] unless two different types of magnetic particles with substantially different magnetic strengths for separation are used (e.g. MACS in combination with Dynabeads) [22].

In the two steps MACS separation we noticed that some CD4+ cells, not being caught in the first isolation column, appeared due to the cascade approach in the second (CD8+) isolation step as contaminants in the CD8+ cell sample. The purity of the sample could be increased by using two-two columns for the two isolation steps. However, this makes MACS slower and more expensive. With pluriSelect the great advantage is that all beads remain on the sieve and non-bound cells are washed through. Hence, the bead recovery is much higher and mutual cross-contaminations are reduced to a minimum.

We noticed slightly lower cell viability after the isolation with pluriSelect system. From our experience with this isolation procedure, we know that the lower viability was due to the shear stress caused by aspirating the cell and bead suspension on a sieve by pipette. This means, the detachment of beads and cells on the sieve by pipetting is a very critical step regarding cell viability and should be performed as gentle as possible. The faster the pipetting, i.e. the higher the shear stress for the cells, the lower is the viability (data not shown). However, the viability level of 90-95% is acceptable [23], and may be further increased by automating the cell separation procedure.

One of the main advantages of the pluriSelect system is its potential to isolate cells directly from the whole blood by positive selection, showing the potential of the method and its robustness. It could be of interest particular for the separation of rare cells where only a few cells could be directly identified in a high blood volume. Nowadays, negative selection methods are preferred for the isolation of rare cells of interest, e.g. erythrocyte rosetting methods by tetramer antibodies [5,6] or magnetic removal of undesired cells [15]. However, this isolation method requires a density gradient centrifugation step too. Application of a density gradient enrichment step can compromise the yield, may lead to additional cell stimulation and affects the detection of cell subtypes with specific density (apoptotic cells). Hence, the isolation of cells directly from the whole blood, without further preparation steps, is preferable. When using pluriSelect, non-target cells (at least non-target in the first isolation step) are not being retained on the first sieve. These remaining cells are washed into a tube from which they could be next cell target and can be tagged and separated by using other antibody-coated beads. These isolation cycles can be repeated many times so that different cell types can be isolated one after another. The fact that the pluriSelect system can be used as cascade isolations, i.e. the simultaneous isolation of different cell types, and the step-by-step isolation cycles makes it an optimal tool to make optimal use of the precious samples, where the source of the sample is limited. Moreover, beads used during isolation can be differentiated among each other and, most important, removed from the cells which means there is no bead contamination in the sample like in MACS. One relevant difference in using whole blood or isolated leukocytes is that in the latter case serum is removed from the cells. This can lead to a difference in yield for those individuals who have a substantial titer of soluble CD4 in their serum and would negatively affect yield with pluriSelect but only little with MACS.

Noteworthy to mention is that cells bound to the beads on a sieve can be lysed directly without using the detachment buffer saving time and the most native conditions of the cells for further molecular analysis on the level of DNA, RNA or proteins. This makes the system extremely versatile for a broad range of applications. If using the positive isolation of cells by the MACS system, the magnetic beads remain on the cell surface. This may affect cell physiology. Even though magnetic beads are regarded to leave cells intact, cells without beads are more attractive for further use. Another example for such bead releasing from the cells is the Dynal system where magnetic Dynabeads are detached from the cell surface via enzymatic reaction [24]. Especially if cells are isolated for clinical, i.e. therapeutic applications the influence of beads on the cell surface can have adverse effects and should be investigated. Reversible staining and isolation technique was developed for isolation of specific T-cells with antitumor activity, where the MHC-StreptagII -StrepTactin binding is completely removed after enrichment by d-biotin molecules [25]. (This system is now commercially available by STAGE Cell Technologies, based on streptamer technology [26]). In future, this method may be combined with the pluriSelect system to reduce stress of cell detachment from the beads.

To sum up, both purity and yield are equivalent for pluriSelect and MACS. Additionally, the cell viability was similar in both systems. Both techniques do not require expensive equipment. However, isolation by the pluriSelect cascade system was much faster than with the MACS system, where two steps procedure is needed for the isolation of two different cell types from one blood sample. Moreover, pluriSelect system allows for the simultaneous sorting of two cell populations which is otherwise only possible by FACS. The fact, that the pluriSelect method can be applied to whole blood or other cellular samples, reduces cell stress and functional impairment otherwise caused by pre-enrichment procedures, etc. In conclusion, the pluriSelect separation system is a versatile, easy to handle and reliable method for cell isolation suitable for a wide range of applications.
